# Supplementary material for: Modeling glioblastoma heterogeneity as a dynamic network of cell states
Source: Mol Syst Biol. 2021 Sep 16;17(9):e10105. doi: 10.15252/msb.202010105 (PMC8444284; doi:10.15252/msb.202010105)
Supplement: Supplementary file 5 — Source Data for Figure 3 [file MSB-17-e10105-s001.zip › Figure3A_sourcedata/GSEA_3065/nosigdb_state6.GseaPreranked.1622718443993/LARSSON_STATE_3.html]

Details for gene set LARSSON\_STATE\_3[GSEA]

|  || Dataset | state6 |
| Phenotype | NoPhenotypeAvailable |
| Upregulated in class | na\_neg |
| GeneSet | LARSSON\_STATE\_3 |
| Enrichment Score (ES) | -0.8381527 |
| Normalized Enrichment Score (NES) | -3.239607 |
| Nominal p-value | 0.0 |
| FDR q-value | 0.0 |
| FWER p-Value | 0.0 |
Table: GSEA Results Summary

  

Fig 1: Enrichment plot: LARSSON\_STATE\_3      
 Profile of the Running ES Score & Positions of GeneSet Members on the Rank Ordered List

  

| PROBE | GENE SYMBOL | GENE\_TITLE | RANK IN GENE LIST | RANK METRIC SCORE | RUNNING ES | CORE ENRICHMENT || 1 | GPNMB |  |  | 1006 | 0.085 | -0.1106 | No |
| 2 | MYH10 |  |  | 1182 | 0.076 | -0.1264 | No |
| 3 | DSEL |  |  | 1304 | 0.071 | -0.1362 | No |
| 4 | SPTBN1 |  |  | 4188 | -0.003 | -0.4670 | No |
| 5 | PLD5 |  |  | 5294 | -0.024 | -0.5925 | No |
| 6 | SCRG1 |  |  | 5450 | -0.027 | -0.6087 | No |
| 7 | MTRNR2L12 |  |  | 6287 | -0.044 | -0.7022 | No |
| 8 | NCAM1 |  |  | 6342 | -0.046 | -0.7058 | No |
| 9 | HSP90AB1 |  |  | 6619 | -0.053 | -0.7345 | No |
| 10 | IQGAP1 |  |  | 6620 | -0.053 | -0.7314 | No |
| 11 | MKI67 |  |  | 6750 | -0.057 | -0.7430 | No |
| 12 | VGF |  |  | 6855 | -0.060 | -0.7515 | No |
| 13 | LAPTM4B |  |  | 6862 | -0.061 | -0.7487 | No |
| 14 | BCAN |  |  | 6866 | -0.061 | -0.7456 | No |
| 15 | FGFBP3 |  |  | 7004 | -0.065 | -0.7576 | No |
| 16 | HNRNPU |  |  | 7074 | -0.067 | -0.7617 | No |
| 17 | GRIA2 |  |  | 7312 | -0.075 | -0.7847 | No |
| 18 | TOP2A |  |  | 7430 | -0.079 | -0.7936 | No |
| 19 | WLS |  |  | 7516 | -0.082 | -0.7987 | No |
| 20 | SYNE2 |  |  | 7598 | -0.086 | -0.8031 | No |
| 21 | SOX6 |  |  | 7616 | -0.087 | -0.8002 | No |
| 22 | ABHD2 |  |  | 7643 | -0.088 | -0.7981 | No |
| 23 | GPM6A |  |  | 7875 | -0.100 | -0.8190 | No |
| 24 | MMP16 |  |  | 7971 | -0.105 | -0.8239 | No |
| 25 | PRKDC |  |  | 8083 | -0.113 | -0.8302 | No |
| 26 | HSP90AA1 |  |  | 8153 | -0.117 | -0.8315 | Yes |
| 27 | SLC44A1 |  |  | 8158 | -0.118 | -0.8253 | Yes |
| 28 | PON2 |  |  | 8177 | -0.120 | -0.8205 | Yes |
| 29 | STT3B |  |  | 8232 | -0.124 | -0.8197 | Yes |
| 30 | NASP |  |  | 8318 | -0.134 | -0.8218 | Yes |
| 31 | ALCAM |  |  | 8323 | -0.135 | -0.8146 | Yes |
| 32 | BSG |  |  | 8443 | -0.151 | -0.8196 | Yes |
| 33 | GPC1 |  |  | 8473 | -0.155 | -0.8141 | Yes |
| 34 | LRPAP1 |  |  | 8498 | -0.161 | -0.8077 | Yes |
| 35 | RTN3 |  |  | 8512 | -0.164 | -0.7999 | Yes |
| 36 | TM9SF3 |  |  | 8539 | -0.170 | -0.7932 | Yes |
| 37 | PGRMC1 |  |  | 8555 | -0.174 | -0.7850 | Yes |
| 38 | HACD3 |  |  | 8558 | -0.175 | -0.7753 | Yes |
| 39 | ATP6AP2 |  |  | 8607 | -0.195 | -0.7697 | Yes |
| 40 | LAMC1 |  |  | 8618 | -0.199 | -0.7596 | Yes |
| 41 | CCDC47 |  |  | 8621 | -0.199 | -0.7485 | Yes |
| 42 | EMP3 |  |  | 8630 | -0.203 | -0.7379 | Yes |
| 43 | SPCS3 |  |  | 8633 | -0.204 | -0.7265 | Yes |
| 44 | LPL |  |  | 8638 | -0.208 | -0.7152 | Yes |
| 45 | DST |  |  | 8644 | -0.211 | -0.7037 | Yes |
| 46 | PDIA3 |  |  | 8647 | -0.212 | -0.6919 | Yes |
| 47 | EMC7 |  |  | 8650 | -0.213 | -0.6800 | Yes |
| 48 | ASPM |  |  | 8657 | -0.215 | -0.6684 | Yes |
| 49 | PRDX4 |  |  | 8660 | -0.217 | -0.6563 | Yes |
| 50 | MANF |  |  | 8665 | -0.220 | -0.6443 | Yes |
| 51 | TMBIM6 |  |  | 8673 | -0.225 | -0.6323 | Yes |
| 52 | FAT1 |  |  | 8679 | -0.228 | -0.6199 | Yes |
| 53 | SSR4 |  |  | 8689 | -0.233 | -0.6077 | Yes |
| 54 | PIF1 |  |  | 8696 | -0.238 | -0.5949 | Yes |
| 55 | GGH |  |  | 8702 | -0.239 | -0.5818 | Yes |
| 56 | CENPF |  |  | 8703 | -0.240 | -0.5682 | Yes |
| 57 | CD320 |  |  | 8704 | -0.241 | -0.5545 | Yes |
| 58 | ITM2B |  |  | 8705 | -0.242 | -0.5407 | Yes |
| 59 | RPN2 |  |  | 8716 | -0.256 | -0.5273 | Yes |
| 60 | B2M |  |  | 8718 | -0.258 | -0.5128 | Yes |
| 61 | FUS |  |  | 8731 | -0.268 | -0.4989 | Yes |
| 62 | CANX |  |  | 8735 | -0.270 | -0.4839 | Yes |
| 63 | KCNQ1OT1 |  |  | 8742 | -0.281 | -0.4686 | Yes |
| 64 | TIMP1 |  |  | 8751 | -0.290 | -0.4531 | Yes |
| 65 | TFPI2 |  |  | 8752 | -0.290 | -0.4366 | Yes |
| 66 | MDM4 |  |  | 8753 | -0.290 | -0.4201 | Yes |
| 67 | APLP2 |  |  | 8755 | -0.292 | -0.4036 | Yes |
| 68 | APP |  |  | 8758 | -0.300 | -0.3867 | Yes |
| 69 | HMGCR |  |  | 8759 | -0.301 | -0.3696 | Yes |
| 70 | DYNC1H1 |  |  | 8761 | -0.302 | -0.3526 | Yes |
| 71 | CD9 |  |  | 8762 | -0.303 | -0.3353 | Yes |
| 72 | SPCS2 |  |  | 8763 | -0.307 | -0.3179 | Yes |
| 73 | TMED9 |  |  | 8765 | -0.311 | -0.3003 | Yes |
| 74 | SQLE |  |  | 8767 | -0.319 | -0.2823 | Yes |
| 75 | PLAT |  |  | 8772 | -0.327 | -0.2641 | Yes |
| 76 | CALR |  |  | 8773 | -0.330 | -0.2453 | Yes |
| 77 | CD63 |  |  | 8776 | -0.335 | -0.2266 | Yes |
| 78 | PTPRZ1 |  |  | 8783 | -0.360 | -0.2068 | Yes |
| 79 | CALU |  |  | 8784 | -0.362 | -0.1862 | Yes |
| 80 | HSPA5 |  |  | 8785 | -0.369 | -0.1652 | Yes |
| 81 | PTN |  |  | 8789 | -0.426 | -0.1413 | Yes |
| 82 | LMAN1 |  |  | 8790 | -0.441 | -0.1163 | Yes |
| 83 | PDIA6 |  |  | 8792 | -0.448 | -0.0909 | Yes |
| 84 | HSP90B1 |  |  | 8794 | -0.487 | -0.0634 | Yes |
| 85 | POSTN |  |  | 8795 | -0.533 | -0.0330 | Yes |
| 86 | MALAT1 |  |  | 8796 | -0.581 | -0.0000 | Yes |
Table: GSEA details [plain text format]

  

Fig 2: LARSSON\_STATE\_3: Random ES distribution      
 Gene set null distribution of ES for **LARSSON\_STATE\_3**
